# Using ChatGPT 4.0 for diagnosis in Dermatology: performance analysis in clinical cases from Anais Brasileiros de Dermatologia^☆^

**DOI:** 10.1016/j.abd.2025.501143

**Published:** 2025-07-03

**Authors:** Matheus Alves Pacheco, Athos Paulo Santos Martini

**Affiliations:** University Hospital, Universidade Federal de Santa Catarina, Florianópolis, SC, Brazil

*Dear Editor,*

Artificial intelligence (AI) has become a topic of growing interest in medical research and is increasingly being applied in dermatology. One of the main branches of AI is Deep Learning, a predominant technology in the processing of complex and high-dimensional data.^1^ Deep Learning uses artificial neural networks that automatically learn the relationships between input data, such as images, and outputs, such as diagnoses, without the need for detailed programming by humans. Inspired by the functioning of the brain, neural networks adjust the intensity of their connections as they learn essential patterns, such as visual characteristics, facilitating the prediction of results.^2^

In this context, ChatGPT is an example of an advanced language model that uses Deep Learning techniques. Belonging to the series of generative pre-training transformer (GPT) models developed by OpenAI, ChatGPT stands out as one of the currently available largest language models, with free public access since 2023.^3^

ChatGPT has already been tested in certificate examinations for different medical specialties, such as ophthalmology (Canada), dermatology (United Kingdom), and in the Title of Specialist in Dermatology (TED) exam in Brazil.^4^, ^5^ In the study that evaluated ChatGPT in TED, the accuracy was 75.34%. Another study in the United Kingdom, with questions from the Specialty Certificate Examination in Dermatology, obtained an accuracy of 63.1% using ChatGPT 3.5, and 90.5% with ChatGPT 4.0.^6^

This study aims to explore the diagnostic performance of ChatGPT in dermatological clinical scenarios published in the “What is your diagnosis?” section of *Anais Brasileiros de Dermatologia* (ABD). A retrospective observational study was conducted to evaluate the performance of ChatGPT 4.0 in dermatological clinical cases published between 2019 and 2023. Cases with complete clinical information, images, laboratory, anatomopathological, and immunohistochemical tests, followed by multiple-choice questions, were included. Cases without multiple-choice questions were excluded.

The interaction with ChatGPT 4.0 followed this sequence: a) Type “I would like you to answer the correct diagnosis of the following clinical case” and press Enter; b) Paste the complete clinical case, including uploaded images and captions; c) Paste the question “What is your diagnosis” and the four alternatives, press Enter; d) Wait for the AI's response and compare it with that of the authors of each case.

The ChatGPT 4.0 responses were compared with the correct option predefined in the ABD, categorizing them as “correct” or “incorrect”. The cases were then classified according to the diagnostic method (clinical, anatomopathological, microbiological). The AI's performance was assessed by the proportion of correct diagnoses in relation to the total number of analyzed cases.

Twenty-five cases were selected, and the AI ​​correctly diagnosed 21, resulting in an accuracy of 84%. Fig. 1 shows the performance of ChatGPT categorized by diagnostic methods, with better performance in cases resolved clinically or by anatomopathological diagnosis and lower accuracy in those that required a microbiological method.

**Figure 1:**
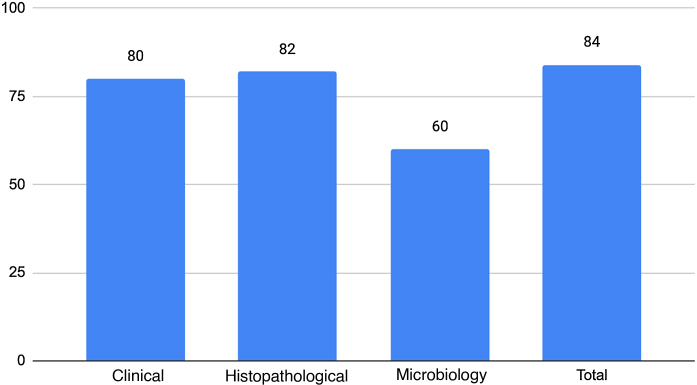
ChatGPT and *Anais Brasileiros de Dermatologia*: ChatGPT performance in different diagnostic methods.

The study assessed the performance of ChatGPT 4.0 in dermatological diagnoses with multiple choice, with four pre-determined options for each clinical case. Unlike a traditional diagnostic accuracy test, in which the AI ​​would provide an open diagnosis, here it selected the correct option among limited alternatives. This does not allow one to state that the diagnostic accuracy of the AI ​​was tested, but rather its performance in a specific context.

Several barriers to the application of AI in dermatology are discussed, including technical issues such as lack of generalizability, standardization of images, and integration of complex clinical data, as well as ethical and regulatory issues such as acceptance of the technology and legal liability in cases of error.^7^

The AI ​​errors in the study were associated with diagnoses involving the integration of clinical, anatomopathological, and microbiological data, suggesting AI limitations in integrating different sources of information in atypical cases.

Therefore, medical practice, especially in a complex specialty such as dermatology, involves a continuous process of learning and improvement, both for human professionals and for artificial intelligence models.

## Financial support

None declared.

## Authors’ contributions

Matheus Alves Pacheco: Design and planning of the study; drafting and editing of the manuscript or critical review of important intellectual content.

Athos Paulo Santos Martini: Drafting and editing of the manuscript or critical review of important intellectual content.

## Conflicts of interest

None declared.

## References

[1] Esteva A., Robicquet A., Ramsundar B., Kuleshov V., DePristo M., Chou K. (2019). A guide to deep learning in healthcare. Nat Med.

[2] Young A.T., Xiong M., Pfau J., Keiser M.J., Wei M.L. (2020). Artificial intelligence in dermatology: a primer. J Invest Dermatol.

[3] Dave T., Athaluri A.S., Singh S. (2023). ChatGPT in medicine: an overview of its applications, advantages, limitations, future prospects, and ethical considerations. Front Artif Intell.

[4] Mihalache A., Popovic M.M., Muni R.H. (2023). Performance of an artificial intelligence chatbot in ophthalmic knowledge assessment. JAMA Ophthalmol.

[5] Jabour T.B.F., Ribeiro JP, Fernandes A.C., Honorato C.M.A., Queiroz MCAP (2024). ChatGPT: performance of artificial intelligence in the dermatology specialty certificate examination. An Bras Dermatol.

[6] Passby L., Jenko N., Wernham A. (2024). Performance of ChatGPT on dermatology specialty certificate examination multiple choice questions. Clin Exp Dermatol.

[7] Gomolin A., Netchiporouk E., Gniadecki R., Litvinov I.V. (2020). Artificial intelligence applications in dermatology: where do we stand?. Front Med (Lausanne).

